# Magnetic Composite Based on Carbon Nanotubes and Deep Eutectic Solvents: Preparation and Its Application for the Determination of Pyrethroids in Tea Drinks

**DOI:** 10.3390/foods12010008

**Published:** 2022-12-20

**Authors:** Xiaodong Huang, Huifang Liu, Xiaomin Xu, Ge Chen, Lingyun Li, Yanguo Zhang, Guangyang Liu, Donghui Xu

**Affiliations:** Institute of Vegetables and Flowers, Chinese Academy of Agricultural Sciences, Key Laboratory of Vegetables Quality and Safety Control, Laboratory of Quality & Safety Risk Assessment for Vegetable Products, Ministry of Agriculture and Rural Affairs of China, Beijing 100081, China

**Keywords:** magnetic solid-phase extraction, deep eutectic solvent, ZIF−8, pyrethroids, tea drinks

## Abstract

In this study, a novel composite material prepared by using deep eutectic solvent (tetrabutylammonium chloride-dodecanol, DES_5_) functionalized magnetic MWCNTs−ZIF−8 (MM/ZIF−8@DES_5_) was employed as an adsorbent for the magnetic solid-phase extraction of six pyrethroids from tea drinks. The characterization results show that MM/ZIF−8@DES_5_ possessed sufficient specific surface area and superparamagnetism, which could facilitate the rapid enrichment of pyrethroids from tea drink samples. The results of the optimization experiment indicated that DES_5_, which comprised tetrabutylammonium chloride and 1-dodecanol, was selected for the next experiment and that the adsorption properties of MM/ZIF−8@DES_5_ were higher than those of MM/ZIF−8 and M-MWCNTs. The validation results show that the method has a wide linear range (0.5–400 μg L^−1^, R^2^ ≥ 0.9905), low LOD (0.08–0.33 μg L^−1^), and good precision (intra-day RSD ≤ 5.6%, inter-day RSD ≤ 8.6%). The method was successfully applied to the determination of pyrethroids in three tea drink samples.

## 1. Introduction

Magnetic solid-phase extraction (MSPE), evolved from traditional solid-phase extraction (SPE), has attracted extensive attention as a preconcentration technique because of its ease of use, low consumption of organic solvents, and time- and cost-efficiency [1]. In a typical MSPE procedure, a small quantity of magnetic sorbent material is directly exposed to a sample solution followed by extraction processing until an adsorption equilibrium is achieved. Then, the sorbents containing the target analytes are retrieved under an external magnetic field; consequently, MSPE exhibits superior extraction efficiency compared with traditional SPE [2]. The sorbent always plays a key role in the MSPE technique, and numerous materials have been prepared as magnetic adsorbents for MSPE, such as carbon nanomaterials, polymers, molecularly-imprinted materials, and porous materials [3,4,5,6].

Multiwalled carbon nanotubes (MWCNTs), a class of tubular carbon nanomaterials based on layers of seamlessly rolled up graphene sheets, have typically been integrated with Fe_3_O_4_ nanoparticles and used as magnetic adsorbents for MSPE. MWCNTs exhibit excellent extraction performance for different analytes due to their remarkable chemical properties, stability, surface area and mechanical strength [7]. In recent years, a new trend has emerged that expands the sample preparation applications of Fe_3_O_4_-MWCNTs (M-MWCNTs) by integrating them with metal–organic frameworks (MOFs) to form functional composites [8].

Zeolitic imidazolate frameworks (ZIFs), as a new family of MOFs, have crystalline three-dimensional frameworks in which transition metal ions (especially as Zn^2+^ Cu^2+^ and Co^2+^) are linked by imidazolate-type organic linkers [9]. ZIFs have found several applications in the adsorption of pollutants from aqueous samples due to their special properties of aqueous and thermal stability, and adsorption capacity [10]. ZIF−8, composed of Zn^2+^ and 2-methylimidazole, has received wide attention in sample preparation, especially for ZIF−8 composites prepared by integration with other functional materials [11]. To promote the extraction performance of ZIF−8 composites for pesticide residues in aqueous samples, deep eutectic solvents (DESs) can be used as an effective reagent to modify ZIF−8 composites [12]. DESs, traditionally prepared by using a hydrogen bond donor (HBD) and hydrogen bond acceptor (HBA) under the action of an intermolecular hydrogen bond, have several fascinating features including low melting point (<100 °C), excellent heat stability, extremely low vapor pressure, naturally degradable, easy to prepare, low cost, and tunable miscibility in water [13,14]. As green and effective reagents, DESs can be selected as an effective extractant in the extraction of active ingredients from plants, heavy metals, environmental pollutants, and pesticides [15,16].

Pyrethroids, as a class of efficient bioderived broad-spectrum insecticides including 42 substances, are the fourth group of insecticides on the basis of the WHO classification [17]. Pyrethroid insecticides have been widely used in agriculture because of their high efficacy, low acute oral toxicity, and harmony with the environment [18,19]. However, long-term and extensive application of pyrethroids could threaten the environment and food chains [20]. Moreover, pyrethroids possess characteristics of bioaccumulation in marine mammals and humans, and their acceptable daily intake values range from 0.02–0.07 mg kg^−1^ day^−1^ and no observed adverse effect level values range from 1–7 mg kg^−1^ day^−1^ [21]. Nowadays, countries in the world have established maximum residue limits (MRLs) of pyrethroids in tea products, for instance MRLs set at 0.1–50 mg kg^−1^ in China (GB 2763 National food safety standard) [22]. Therefore, it is necessary to establish a sensitive and reliable determination method for monitoring pyrethroid pesticides.

This study aimed to prepare a magnetic composite based on carbon nanotubes and deep eutectic solvents and employ this as an adsorbent for the MSPE of pyrethroids from tea (*Camellia sinensis* L.) drink samples. The modification of M-MWCNTs/ZIF−8 (MM/ZIF−8) by the use of ionic liquids and the functionalization of MOFs by the introduction of DESs had been reported. However, there are no reports of tetrabutylammonium chloride-dodecanol-based DES_5_ being used to functionalize a MM/ZIF−8 composite or their use as an adsorbent for the MSPE of pyrethroids. The MM/ZIF−8@DES was obtained and characterized, and the pretreatment technique parameters were optimized. Finally, the established MM/ZIF−8@DES-based sample pretreatment technique was successfully used to extract and determine the amounts of six pyrethroids in tea drinks.

## 2. Materials and Methods

### 2.1. Preparation of Magnetic Materials

#### 2.1.1. Preparation of MM/ZIF−8

M-MWCNTs were prepared following a modified chemical co-precipitation method reported previously [23]. First, 0.2 g of MWCNTs powder (95%, inside diameter of 3–5 nm, length of 50 μm, Aladdin Co., Shanghai, China) was suspended in ultrapure water (240 mL) in a three-necked flask under ultrasonic irradiation for 1 h. After that, 1.8 g of FeCl_3_·6H_2_O (Aladdin Co., Shanghai, China) and 0.8 g of FeCl_2_·4H_2_O (Aladdin Co., Shanghai, China) were added to the flask with mechanical stirring for 30 min at 80 °C. Then, 10 mL of NH_3_·H_2_O (28%, Aladdin Co., Shanghai, China) was slowly transferred into the flask, followed by another 30 min of incubating. Finally, the M-MWCNT material was gathered by magnetic adsorption and cleaned for 3 rounds to remove unreacted chemicals with ultrapure water and anhydrous ethanol (analytical grade, Beijing Bailingwei Science and Technology Co., Beijing, China), sequentially.

The preparation of MM/ZIF−8 was conducted according to our previously published method [24]. First, all of the obtained M-MWCNTs from the former step were suspended in anhydrous ethanol (140 mL), which contained 230 µL of mercaptoacetic acid (analytical grade, Beijing Bailingwei Science and Technology Co., Beijing, China), followed by mechanical stirring for 60 min at room temperature. Then, the reacted mixture was separated with the help of an external magnet and cleaned for 3 rounds to remove unreacted chemicals with ultrapure water and anhydrous ethanol, sequentially. After that, the synthetic product was added into the mixture of anhydrous ethanol-ultrapure water (1:1, v/v, 240 mL), which also contained 0.26 g of ZnSO_4_·7H_2_O (Aladdin Co., Shanghai, China), and the mixture was stirred for 1.5 h. Then, 0.84 g of 2-methylimidazole (Aladdin Co., Shanghai, China) was dissolved in anhydrous ethanol (20 mL) and transferred into the above mixture, followed by another 8 h of mechanical stirring. Finally, the obtained synthetic products were acquired by means of magnetic adsorption and cleaned for several rounds to remove unreacted chemicals using anhydrous ethanol and ultrapure water, sequentially. The synthetic MM/ZIF−8 was dried in a vacuum drying oven at 60 °C for a whole day.

#### 2.1.2. Preparation of MM/ZIF−8@DES

The preparation of six kinds of DESs were carried out as follows: HBAs and HBDs were blended in a 1:2 molar ratio, followed by constant mechanical stirring at 40 °C until a homogeneous solution was formed. The prepared DESs products were based on two kinds of quaternary ammonium salts and three different HBDs according to a specific molar ratio (Table 1).

MM/ZIF−8@DES was fabricated in accordance with a published method with a slight modification [25]. First, Different amounts of MM/ZIF−8 (0.25, 0.4, 0.5, 0.75, 1.0, and 1.25 g) were added to the 4 mL of methanol (HPLC grade, Sigma-Aldrich, STL, USA) solution, which including 0.5 g of DES. Then, the mixtures were incubated for 1 h under ultrasonic conditions at ambient temperature. After that, the synthetic MM/ZIF−8@DES was obtained via magnetic adsorption, followed by 3 rounds of anhydrous ethanol washes, and vacuum drying at 60 °C for a whole day.

### 2.2. The MSPE Procedure

A total of 100 mg L^−1^ of stock standard solution was prepared as follows: First, 1 mL of pyrethroid standard solutions (1000 mg L^−1^, Agro-Environmental Protection Institute, Tianjin, China) of cyhalothrin, cyfluthrin, cypermethrin, flucythrinate, fenvalerate and fluvalinate, were added into a 10 mL volumetric flask, respectively. After that, the mixture was diluted with HPLC grade methanol (Sigma-Aldrich, STL, USA) to volume followed by hand shaking for 20 s. Then, the prepared stock solution was transferred into a brown color screw sample bottle and stored at −20 °C.

**Figure 1:**
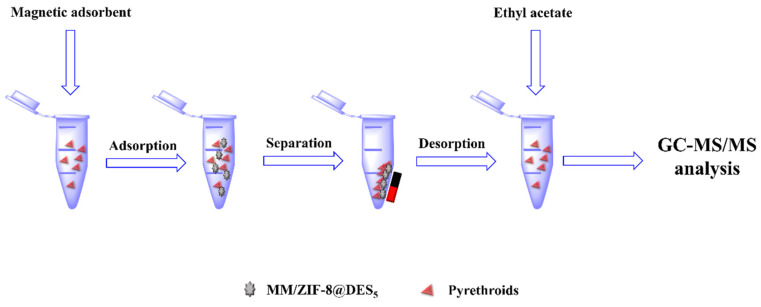
Schematic procedure for the proposed MSPE method.

The MSPE was programed as follows (Figure 1): First, 6 mg of MM/ZIF−8@DES_5_ was suspended into 5 mL of sample solution, which contained a certain concentration of pyrethroids, followed by 10 min of shaking to reach an adsorption equilibrium state. Then, the mixed solution was placed in an outside magnetic field to remove the supernatant. After that, 3 min of vortex desorption was carried out by adding 3 mL of ethyl acetate (HPLC grade, Sigma-Aldrich, STL, USA) to the adsorbent. The supernatant desorption solution was collected by an outside magnetic field, followed by an evaporation treatment until dry via gentle nitrogen-blowing at 40 °C. Finally, the residue component was dissolved once again with 0.5 mL of acetone (HPLC grade, Sigma-Aldrich, STL, USA), a 1.0 μL portion of which was analyzed by gas chromatography-tandem mass spectrometry (GC-MS/MS, model GCMS-TQ 8040, Shimadzu, Kyoto, Japan). The operational parameters for GC-MS/MS and the detailed multiple reaction monitoring (MRM) transitions for the six pyrethroids are presented in Table 2 and Table 3, respectively.

## 3. Results and Discussions

### 3.1. Characterization of Magnetic Materials

**Figure 2:**
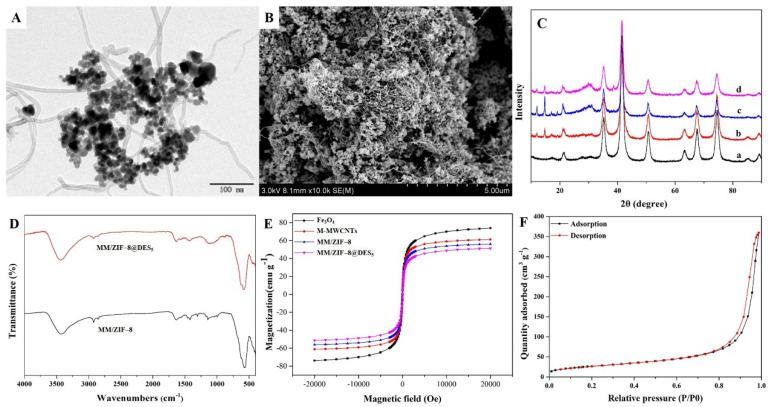
Characterization of the prepared materials: (**A**) TEM image of M-MWCNTs; (**B**) SEM image of MM/ZIF−8@DES_5_; (**C**) XRD patterns of (a) Fe_3_O_4_, (b) M-MWCNTs, (c) MM/ZIF−8, and (d) MM/ZIF−8@DES_5_; (**D**) FT-IR spectra of magnetic composites; (**E**) Magnetic curves of obtained materials; (**F**) N_2_ adsorption-desorption isotherms (obtained at 300 K) of MM/ZIF−8@DES_5_.

The morphologies of M-MWCNTs and MM/ZIF−8@DES_5_ were studied by transmission electron microscopy (TEM, performed with JEM-200CX transmission electron microscope, JEOL, Tokyo, Japan) and scanning electron microscopy (SEM, performed with JSM-6300 scanning electron microscope, JEOL, Tokyo, Japan), respectively. As shown in Figure 2A, MWCNTs present a typical tubular shape, and Fe_3_O_4_ nanoparticles were adhered to the surface of MWCNTs with slight agglomeration. As shown in Figure 2B, MM/ZIF−8@DES_5_ reveals a highly-porous block-shaped structure with a rough surface, which indicates a good adsorption prospect for the enrichment of pesticides. The crystal structures of the obtained materials were identified by X-ray diffractometer (XRD, performed with D8 Advance X-ray powder diffractometer, Bruker, Karlsruhe, Germany), and the results are shown in Figure 2C. Several primary diffraction peaks for Fe_3_O_4_, appearing at 21.3°, 35.2°, 41.5°, 63.2°, 67.4°, and 74.5°, can be seen clearly in three M-MWCNTs-based materials. These results indicated that Fe_3_O_4_ nanoparticles were retained during the formation of the composite materials. Peaks of 10–25° and 28–34° demonstrated that the MM/ZIF−8@DES_5_ was successfully prepared [26].

Figure 2D exhibits the Fourier transform infrared (FT-IR, performed with FT-IR-8400 spectrometer, Shimadzu, Kyoto, Japan) spectra of the obtained products. The adsorption bands at 572 cm^−1^ were originated from Fe-O stretching vibration, indicating that the successful encapsulation of Fe_3_O_4_ into magnetic materials. Meanwhile, the adsorption peak at 1534 cm^−1^ corresponds to the cylinder-like carbon structure of MWCNTs, and the characteristic bands at 432, 811–1360, and 1418 cm^−1^ probably because of the Zn-N stretching vibration and at 911 cm^−1^ for the C-N stretching vibration from the imidazole ring. These results suggest the successful preparation of MM/ZIF−8 [23]. The peak at 1475 cm^−1^ can be attributed to the C-N stretching vibration of DES [27]. Furthermore, the characteristic absorption peaks of ZIF−8 in the spectra of MM/ZIF−8@DES_5_ were weaker than those in MM/ZIF−8, which affected the FT-IR spectral scanning of ZIF−8 because of the coating of DES. The above results indicate that MM/ZIF−8@DES_5_ was successfully synthesized.

Magnetic characteristics of the prepared magnetic composites were confirmed by vibrating sample magnetometry (VSM, performed with 7410 magnetometer, Lake Shore, Columbus, USA) (Figure 2E). The magnetic hysteresis loops of Fe_3_O_4_, M-MWCNTs, MM/ZIF−8, and MM/ZIF−8@DES_5_ all presented an S-like curve, and the saturation magnetizations of them were 75.5, 61.3, 56.1, and 51.3 emu g^−1^, respectively. Moreover, values of coercivity and remanence for the above materials are negligible. These results show that all prepared materials are superparamagnetic and are capable of rapidly separating with an external magnet [12].

The porosity of MM/ZIF−8@DES_5_ was investigated by an N_2_ adsorption-desorption isotherm (performed with ASAP 2020 surface area and porosity analyzer at 300 K, Micromeritics, Norcross, USA). As presented in Figure 2F, the comprehensive pattern of curves reveals that the N_2_ adsorption increased slightly at low relative pressures and sharply increased at high relative pressure. These results suggest that the pore size of MM/ZIF−8@DES_5_ is in the range of the mesoporous to microporous scale [24]. Furthermore, the Brunauer–Emmett–Teller surface area and the pore volume were 133.68 m^2^ g^−1^ and 0.574 mL g^−1^, respectively. All the above results illustrate that MM/ZIF−8@DES_5_ possesses an acceptable surface area and total volume, which are conducive to the adsorption of pyrethroids.

### 3.2. The Optimization of the MSPE Parameters

To achieve a satisfactory extraction efficiency for the developed MSPE technique, a single-factor experimental design was adopted to optimize the type and quantity of DES, and the adsorption and desorption conditions.

#### 3.2.1. Selection of Type and Quantity of DES_5_

**Figure 3:**
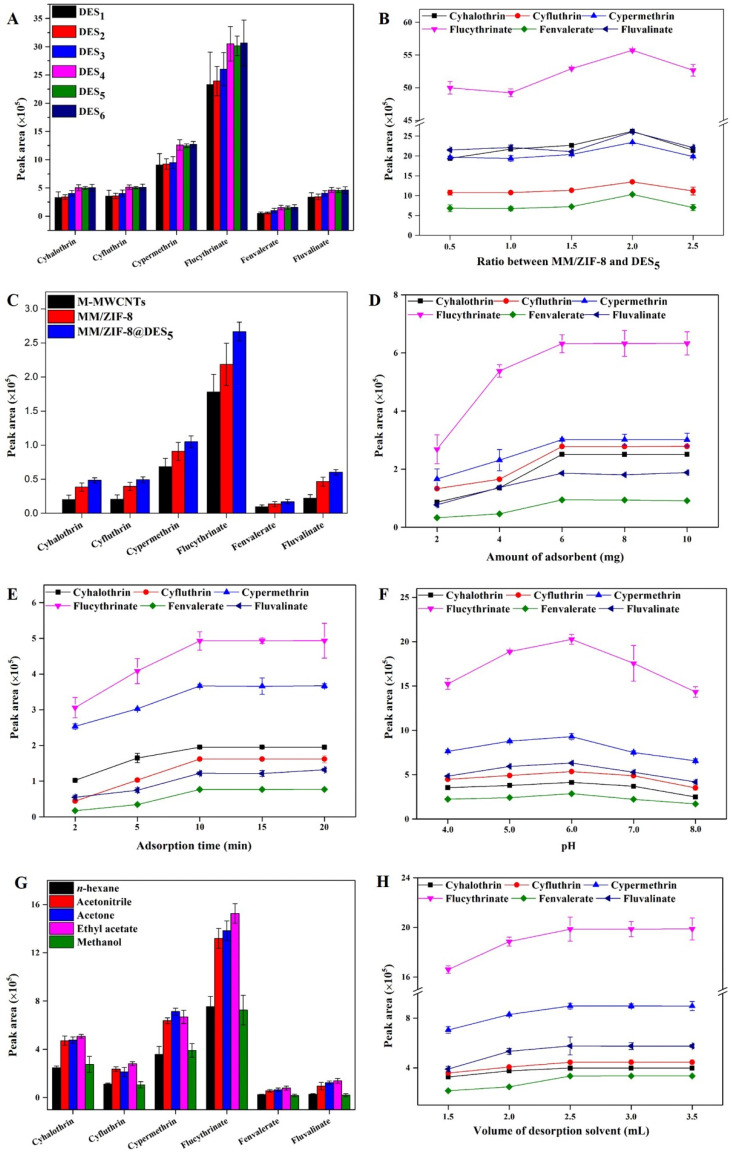
Effect of conditions on the MSPE of pyrethroids: (**A**) type of DES; (**B**) mass ratio of MM/ZIF−8, and DES_5_; (**C**) type of adsorbent; (**D**) amount of adsorbent; (**E**) adsorption time; (**F**) pH value of sample solution; (**G**) type of desorption solvent; (**H**) desorption time.

To examine the effect of different DESs on the extraction performance, six types of DES with different HBDs and HBAs were prepared and used to coat the surface of the magnetic composites. As shown in Figure 3A, the extraction effects of DES_4_, DES_5_, and DES_6_ were more superior to those of DES_1_, DES_2_, and DES_3_. In DES_1_, DES_2_, and DES_3_, the extraction performance for pyrethroids promoted with the alkyl chain length of the HBD increased, which was ascribed to the principle of similar phase-dissolving. In DES_4_, DES_5_, and DES_6_, the extraction performance was not related to the length of the alkyl chain of the HBD. With consideration of the smallest relative standard deviations (RSD), the optimal modifier chosen was DES_5_.

The mass ratio of MM/ZIF−8 to DES has a significant influence on the adsorption of the analytes. To achieve a positive extraction efficiency, several mass ratios (0.5:1, 1:1, 1.5:1, 2:1, and 2.5:1) of MM/ZIF−8 to DES_5_ were investigated (Figure 3B). The extraction performance for analytes were optimal when the mass ratio was 2:1. Therefore, this ratio was selected as the optimal constituent of MM/ZIF−8@DES_5_.

#### 3.2.2. Effect of Type and Dosage of Adsorbent

To identify the extraction performance of prepared magnetic materials for pyrethroids, three composites, including MM/ZIF−8@DES_5_, MM/ZIF−8, and M-MWCNTs were selected as potential magnetic adsorbents for the MSPE procedure (Figure 3C). Clearly, MM/ZIF−8@DES_5_ exhibits the optimum extraction performance for pyrethroids, while the MM/ZIF−8 was the second best and M-MWCNTs was the worst. The possible reason could be that more surface area is made available on the M-MWCNTs after the formation of MM/ZIF−8, which is then available for the adsorption of target analytes. Furthermore, the hydrophobic nature and abundance of potential hydrogen bond donors and acceptors of the DES on MM/ZIF−8@DES_5_ could facilitate its adsorption for pyrethroids. Therefore, MM/ZIF−8@DES_5_ was chosen as the magnetic adsorbent in the MSPE procedure being developed.

The quantity of the sorbent can obviously affect the extraction performance of the MSPE procedure. To achieve a favorable extraction performance for pyrethroids, 2, 4, 6, 8 and 10 mg of magnetic sorbent were suspended separately to the fortified sample solutions (50 µg L^−1^). As shown in Figure 3D, 6 mg of adsorbent gives an outstanding extraction performance for pyrethroids and was consequently set as the optimum dosage of the adsorbent.

#### 3.2.3. Effect of Extraction Time

To study the effect of the extraction time on MSPE performance, 2 to 20 min of shaking time was tested. The extraction efficiencies were promoted with the increase of treatment time from 2 to 10 min. After that, the extraction efficiencies were maintained relatively unchanged as the treatment time increased from 10 to 20 min (Figure 3E). Therefore, the adsorption time was set at 10 min.

#### 3.2.4. Effect of pH of Sample Solution

Solution pH is another crucial parameter for the optimization of the MSPE procedure because of its probability of changing the surface charge of the magnetic adsorbent and/or chemical form of the analyte. Therefore, sample solutions with a pH ranging from 4.0 to 8.0 were prepared by adjusting with HCl or NaOH as necessary. As shown in Figure 3F, the extraction performance for six analytes were slightly promoted with the increase of pH value from 4.0 to 6.0, whereas they were reduced when the pH value increased past 7.0. The probable cause was that alkaline conditions affect the stability of pyrethroids. Furthermore, the pH values for most of the tea drinks were approximately 5.6–5.8 [28]. Therefore, the tea drink samples needed no adjustment.

#### 3.2.5. Selection of the Type and Volume of Desorption Solvent

The type and usage of a desorption solvent played a key role in achieving a favorable recovery of the target analytes. To study the affection of eluent on desorption performance, HPLC grade acetonitrile, methanol, acetone, n-hexane, and ethyl acetate were selected as the potential desorption solvents for this study, and the results indicated that the ethyl acetate gave the optimum desorption performance (Figure 3G). All candidate desorption solvents were obtained from Sigma-Aldrich (ST, USA). Meanwhile, the volume of eluent was optimized from 1.5 to 3.5 mL, and the results illustrated that 2.5 mL of ethyl acetate was adequate for the desorption process (Figure 3H). Hence, 2.5 mL of ethyl acetate was selected as the ideal desorption conditions in further studies.

### 3.3. Method Validation

To confirm the performance of the established method, different parameters of linearity, limit of detection (LOD), and precision were investigated by analyzing fortified blank samples (Table 4). The linear range of the as-developed method was acquired by analyzing working solutions containing six pyrethroids (0.5, 1, 2, 5, 10, 20, 50, 100, 200, and 500 μg L^−1^) and plotting the concentration versus the peak area. The results suggested good linearity for the six pyrethroids with the correlation coefficient (R^2^) ranging from 0.9905 to 0.9925. The LODs were derived from the signal-to-noise ratio (S/N) of 3, and the results are 0.08–0.33 μg L^−1^. The precision of the developed method was studied by measuring the intra- and inter-day RSDs, and the RSD values were less than 5.58% and 8.58%, respectively. All the above results suggested the established method possesses a prospective performance for the determination of pyrethroid pesticide residues in tea drinks.

### 3.4. Comparison of the Proposed MSPE with Other Published Methods

To demonstrate the potential application of MM/ZIF−8@DES_5_ as an adsorbent in MSPE, the as-developed method was compared with several published methods (Table 5). After careful consideration of the exhibited parameters, the proposed method showed a similar performance to those previously reported.

### 3.5. Real Sample Analysis

Under the optimal conditions, the as-developed method was used to determine six pyrethroid residues in three tea drink samples (red tea, green tea, and oolong tea, purchased from a local market), and negative results of pyrethroid residues were obtained; due to the response values of the MS instrument for analytes, which were lower than the LODs (Table 6). To confirm the adaptability of the proposed method for real tea drink samples, standard solutions at 10 and 100 μg L^−1^ were spiked in real samples and investigated to accompany the real sample analysis. The recovery experiments were performed in triplicate. The recovery results suggested that the established method exhibits a satisfactory performance for the determination of pyrethroid residues in tea drinks.

## 4. Conclusions

In this study, a novel and efficient DES-type surfactant functionalized MM/ZIF−8 composite (MM/ZIF−8@DES_5_) was successfully prepared and selected as an effective magnetic adsorbent for the determination of pyrethroids in tea drink samples. Material characterization indicated that the MM/ZIF−8@DES_5_ possesses a sufficient specific surface area, a decent pore volume, and superparamagnetism, which will enable the rapid separation of pyrethroids from tea drink samples. Validation of the proposed method suggested excellent linearity, low LODs, and good precision. The developed method possesses a considerable future for the monitoring of organic pollutants in the environment or in food samples.

## Figures and Tables

**Table 1:** List of synthetic components of different DESs.

DES	HBA	HBD	Molar Ratio of HBA to HBD
DES_1_	1-methyl-3-octyl imidazolium chloride ^a^	1-undecanol ^b^	1:2
DES_2_	1-methyl-3-octyl imidazolium chloride	1-dodecanol ^c^	1:2
DES_3_	1-methyl-3-octyl imidazolium chloride	1-tridecanol ^d^	1:2
DES_4_	tetramethylammonium chloride ^e^	1-undecanol	1:2
DES_5_	tetramethylammonium chloride	1-dodecanol	1:2
DES_6_	tetramethylammonium chloride	1-tridecanol	1:2

^a,b,c,d,e^ Five kinds of reagents were analytical grade; obtained from Beijing Bailingwei Science and Technology Co. (Beijing, China).

**Table 2:** Operational parameters for GC-MS/MS.

GC Specification		MS Specification	
Column	Rtx-5Ms capillary column (0.25 mm (id) × 30 m, 0.25 μm film thickness, Restek, Bellefonte, PA, USA)	Interface temperature	300 °C
Column oven temp	40 °C (4 min), 40–125 °C at 25 °C min^−1^, 125–300 °C at 10 °C min^−1^, and finally held for 6 min. The total run time was 21 min	Ion source temperature	200 °C
Carrier gas and column flow	Helium flow rate = 1.0 mL min^−1^	Measurement mode	MRM
Injection	1.0 μL, splitless mode	—	—

**Table 3:** Acquisition and chromatographic parameters of the six pyrethroids.

Analytes	t_R_ (min)	MRM1 (*m/z*)	CE1 (eV)	MRM2 (*m/z*)	CE2 (eV)
Cyhalothrin-1	18.785	197.0 > 161.0	8	197.0 > 141.0	12
Cyhalothrin-2	18.962	197.0 > 161.0	8	197.0 > 141.0	12
Cyfluthrin-1	20.304	226.1 > 206.1	14	226.1 > 199.1	6
Cyfluthrin-2	20.398	226.1 > 206.1	14	226.1 > 199.1	6
Cyfluthrin-3	20.461	226.1 > 206.1	14	226.1 > 199.1	6
Cyfluthrin-4	20.501	226.1 > 206.1	14	226.1 > 199.1	6
Cypermethrin-1	20.630	163.1 > 127.1	6	163.1 > 91.0	14
Cypermethrin-2	20.733	163.1 > 127.1	6	163.1 > 91.0	14
Cypermethrin-3	20.793	163.1 > 127.1	6	163.1 > 91.0	14
Cypermethrin-4	20.831	163.1 > 127.1	6	163.1 > 91.0	14
Flucythrinate-1	20.794	199.1 > 157.1	10	199.1 > 107.1	22
Flucythrinate-2	20.985	199.1 > 157.1	10	199.1 > 107.1	22
Fenvalerate-1	21.430	419.1 > 225.1	6	419.1 > 167.1	12
Fenvalerate-2	21.640	419.1 > 225.1	6	419.1 > 167.1	12
Fluvalinate-1	21.540	250.1 > 55.0	20	250.1 > 200.0	20
Fluvalinate-2	21.600	250.1 > 55.0	20	250.1 > 200.0	20

**Table 4:** Analytical parameters of MM/ZIF−8@DES_5_–MSPE-GC-MS/MS method for the analysis of the six pyrethroids from tea drinks.

Analytes	Calibration	Linear Range (μg L^−1^)	R^2^	Intraday RSD (%)	Interday RSD (%)	LOD (μg L^−1^)
Cyhalothrin	y = 3703.2x −46478	0.5–400	0.9905	4.00	7.57	0.08
Cyfluthrin	y = 3791.2x −47015	0.5–400	0.9907	4.84	7.56	0.33
Cypermethrin	y = 8168.8x −92816	0.5–400	0.9925	2.99	5.77	0.22
Flucythrinate	y = 19644.0x −251300	0.5–400	0.9910	3.50	5.83	0.13
Fenvalerate	y = 3037.9x −38641	0.5–400	0.9912	4.83	7.66	0.24
Fluvalinate	y = 5608.7x −59333	0.5–400	0.9922	5.58	8.58	0.10

**Table 5:** Comparison of the developed pretreatment technique with other published methods.

Method	Sorbent	Sample Amount (mL)	Sorbent Amount (mg)	Extraction Time (min)	Volume of Eluent (mL)	Linear Range (μg L^−1^)	LOD (µg L^−1^)	Ref.
MSPE-DLLMESFO- GC-ECD	Fe_3_O_4_/MIL-101(Cr)	50	10	10	methanol, 0.4	0.05–10	0.008–0.015	[29]
MPSE-GC	Magnetic silica aerogels	2.5	30	10	ethyl acetate	0.04–8	0.008–0.024	[30]
dSPE-UFLC-UV	Fe_3_O_4_/C/PANI microbowls	150	8	12	methanol, 3	0.1–20	0.025–0.032	[31]
MSPE-HPLC-UV	Fe_3_O_4_-MCNTs	10	40	15	5% acetic acid acetonitrile, 3	0.05–25 μg g^−1^	0.010–0.018 μg g^−1^	[32]
MSPE-GC-MS/MS	MM/ZIF−8@DES	5	6	10	ethyl acetate, 3	0.5–400	0.08–0.33	This work

**Table 6:** Analytical results for the determination of pyrethroids in real tea drink samples.

Matrix	Analyte	Spiked Levels (μg L^−1^, *n* = 3)				
		0	10		100	
		Found	Recovery (%)	RSD (%)	Recovery (%)	RSD (%)
Red tea	Cyhalothrin	<LOD	72.5	7.1	85.4	2.5
	Cyfluthrin	<LOD	76.3	3.2	86.1	2.8
	Cypermethrin	<LOD	77.5	6.6	82.5	5.3
	Flucythrinate	<LOD	70.4	6.2	87.7	2.1
	Fenvalerate	<LOD	76.1	3.4	87.2	3.6
	Fluvalinate	<LOD	78.3	4.9	96.4	4.3
Green	Cyhalothrin	<LOD	83.7	6.0	86.0	4.1
tea	Cyfluthrin	<LOD	75.2	5.1	84.0	1.9
	Cypermethrin	<LOD	79.1	4.6	82.2	2.1
	Flucythrinate	<LOD	77.3	6.8	89.3	3.5
	Fenvalerate	<LOD	72.8	8.7	87.4	3.1
	Fluvalinate	<LOD	80.7	7.9	86.3	4.5
Oolong	Cyhalothrin	<LOD	74.4	7.1	86.0	2.4
tea	Cyfluthrin	<LOD	75.2	6.8	83.8	2.9
	Cypermethrin	<LOD	72.3	10.0	88.8	7.6
	Flucythrinate	<LOD	73.6	3.7	93.1	3.9
	Fenvalerate	<LOD	81.1	5.3	91.8	1.9
	Fluvalinate	<LOD	82.6	7.9	86.3	4.5

## Data Availability

The data presented in this study are available on request from the corresponding author.
